# Safety and efficacy of Intraurethral Mitomycin C Hydrogel for prevention of post-traumatic anterior urethral stricture recurrence after internal urethrotomy

**DOI:** 10.5249/jivr.v8i2.812

**Published:** 2016-07

**Authors:** Mahmoudreza Moradi, Katayoun Derakhshandeh, Babak Karimian, Mahtab Fasihi

**Affiliations:** ^*a*^Department of Urology, Imam Reza Hospital, Kermanshah University of Medical Sciences, Kermanshah, Iran.; ^*b*^Tissue Engineering & Regenerative Medicine (TERM) Research Center, KUMS, Kermanshah, Iran.; ^*c*^School of Pharmacy, Kermanshah University of Medical Sciences, Kermanshah, Iran.

## Abstract

**Background:**

Evaluation of the safety and efficacy of intraurethral Mitomycin C (MMC) hydrogel for prevention of post-traumatic anterior urethral stricture recurrence after internal urethrotomy.

**Methods:**

A thermoresponsive hydrogel base consisting of 0.8 mg MMC with 1cc water and propylene glycol to PF-127 poloxamer was used in theater. 40 male patients with short, non-obliterated, urethral stricture were randomized into 2 groups: control and MMC. After internal urethrotomy, the MMC group patients received the MMC-Hydrogel while the others were just catheterized. Both groups had their catheters for at least 1 week. After surgery, they were followed up by means of medical history and physical examination, monitoring voiding patterns and retrograde urethrogram at 1 month, 6 months and 1 year after surgery.

**Results:**

40 male patients between 14 to 89 years old (Mean = 54.15) underwent internal urethrotomy. The average age for the control and MMC group was 54.55±21.25 and 53.75±24.75 respectively. In a comparison of age between the two groups, they were matched (P=0.574). Stricture length was 10.7±5.9 and 9.55±4.15 mm for the control and MMC group respectively. There were no statistically meaningful differences between the two groups (P=0.485). Fifteen patients had a history of one previous internal urethrotomy which in a comparison between the two groups meant there was no meaningful difference (P = 0.327). During postoperative follow up, total urethral stricture recurrence happened in 12 patients: 10 patients (50%) in control group and 2 patients (10%) in MMC group. The difference was statistically significant (P = 0.001). There were no significant complications associated with the MMC injection in our patients.

**Conclusions:**

Based on our results, MMC Hydrogel may have an anti-fibrotic action preventing post-traumatic anterior urethral stricture recurrence with no side effects on pre-urethral tissue. Due to our study limitations, our follow up time and the small number of patients, our results were not conclusive and further studies will be needed with a longer follow up time.

## Introduction

Urethral stricture is a troublesome complication in male patients and causes a great morbidity and considerable health-related costs. It is anatomically classified into two forms: anterior and posterior urethral strictures, of which anterior urethral strictures are the most common.

Various therapeutic methods have been utilized for treatment of anterior urethral strictures, including: urethral dilatation, internal urethrotomy and different types of urethroplasty. One of the most used methods is internal urethrotomy, in which the fibrotic stricture is incised with a cold knife endoscopically. Unfortunately, the effectiveness of internal urethrotomy is low, and has been evaluated to be as low as 25% to 75% in different studies depending on patients` situation, the length and depth of stricture, previous history of internal urethrotomy and recurrence of stricture.^1,2^ Therefore any procedure that is able to diminish the rate of stricture or its recurrence would warrant further investigation.

Surprisingly, urology reference books do not mention the use of chemicals for the prevention of urethral stricture or fibrosis. Of course there are a few reports of local injection of Triamcinolone between 1965 to 1975 with little success, and one study with good clinical results in 2010 and scattered mentions of gene therapy.^3,4^

It has been scientifically demonstrated that Mitomycin C has anticollagenic and antifibroblastic effects. This characteristic of MMC has been introduced in different reports, for example it has been used under the scleral flap during trabeculectomy, for post-traumatic subglottic scar, or in myringotomy for otitis media.^5,6,7,8,9,10,11^

The therapeutic effectiveness of MMC for prevention of urethral stricture after internal urethrotomy was reported in one randomized clinical trial, where MMC was injected to the fibrotic site directly with good clinical results.^12^ However, owing to the possible complication of extensive extravasations of MMC through the spongiosum and into the venous drainage and a relatively difficult procedure, ^3^ we were encouraged to look for a novel way to apply MMC locally with the best efficacy and as few complications as possible. 

As far as we know, our study is the first of this kind, using a hydrogel-based form of MMC in the prevention of anterior urethral stricture. We have used hydrogel in our drug formulation to accomplish a more appropriate form of the drug with better effects and minimizing the side-effects of direct injection. We are convinced that if this study shows a meaningful effect on lowering the rate of stricture recurrence after internal urethrotomy, it will probably open a new horizon in urethral stricture recurrence management. 

## Methods

Patients with non-obliterated, anterior urethral stricture with a length of less than 15 mm and no or mild spongiofibrosis in ultrasonography who were candidates for internal urethrotomy were included in our study. Patients with a history of open urethroplasty, or who had had internal urethrotomy on more than one occasion, who suffered from severe or obliterated stenosis and pediatric patients were excluded from the study. For each patient, history was taken and physical examination was done and routine lab tests like fasting blood sugar, blood urea and creatinine and urine culture were examined. Patients with positive urine culture were put on suitable treatment and after their culture became negative entered the study.

For all the patients, retrograde urethrogram and ultrasonography of urethra were taken before the procedure, and length of stenosis and existence of spongiofibrosis were evaluated. Then patients were randomized to two groups: Control and MMC group.

For all patients, the procedures were done under spinal or general anesthesia and by one surgeon. As a routine internal urethrotomy, the fibrosis site of stricture was incised transurethrally at XII o clock via cold knife urethrotomy. In the control group no drug was injected after internal urethrotomy and only a Foley catheter was inserted. In the MMC group, after internal urethrotomy, first of all the distance between the stricture and the meatus was measured in the retrograde urethrogram and also during Cystourethroscopy and then a Foley catheter was fixed and put under traction, so the bladder neck was closed. Then from the side of the Foley catheter, a small feeding tube was entered through the urethra to the same distance measured before, so that it reached the site of the stricture and the hydrogel-based MMC was injected through the feeding tube. Finally the distal part of the urethra was clamped for six hours to prevent the jelly from being expelled. The Foley catheter was fixed for one week for both groups of patients. 

After surgery, they were followed with medical history and physical examination with focus on voiding patterns, and a retrograde urethrogram at 1 month, 6 months and 1 year after surgery. Any symptoms or sign of possible complication were recorded for every patient.

If a patient had no complaints of irritative or obstructive symptoms or post voiding residue, on history or physical examination and no sign of any obvious stricture at retrograde urethrography, it was considered to be therapeutically successful.

The preparation of the drug formulation was done one day before surgery, at the pharmaceutics lab by the pharmaceutics expert: a sufficient amount of poloxamer polymer (PF-127)^13^ was added to the rotating 4°C distilled water and then kept in a refrigerator for 24 hours (Cold method). Then the mixture was brought to theatre on the day of surgery and 0.8 mg MMC and 1 cc mixture of distilled water and propylene glycol in a 40/60 relation were added to it, and handed to the surgeon. This formulation was a thermosensitive compound; somehow it has a solution shape at 4°Ctemperature and a gel shape at body temperature. So, because of this rapid change in condition, the surgeon had to inject the medication quickly.

The information before and after the procedure was recorded in the special check lists. Finally, data were analyzed by SPSS 16. In order to match the age and compare the stricture lengths, independent t-test and U-man and Tiny test were used. In order to match the history of surgery and compare stricture recurrence, K^2^ (chi square) and Fischer tests were used.

We declare that this study has been approved by Kermanshah University of Medical Sciences Medical Ethics Committee and we have counseled all of our patients and have received written consent from them.

## Results

Between July 2009 to August 2010, 40 male patients between 14 to 89 years old (Mean = 54.15) underwent internal urethrotomy. The average age for the control and MMC group was 54.55±21.25 and 53.75±24.75 respectively. In an age comparison of between the two groups, they were matched (P=0.574). Stricture length was 10.7±5.9 and 9.55±4.15 mm for control and MMC group respectively. There were no statistically meaningful differences between the two groups (P=0.485). History of one previous internal urethrotomy existed in 15 patients which means that in a comparison between the two groups there was no meaningful difference (P = 0.327).

During postoperative follow up, total urethral stricture recurrence happened in 12 patients: 10 patients (50%) in the control group and 2 patients (10%) in the MMC group. The difference was statistically significant (P = 0.001). There were no significant complications associated with MMC injection in our patients. 

## Discussion

Although history of urethral stricture treatment is as old as the creation of urology itself, essential developments in its treatment have been made in the past 50 years, mainly internal urethrotomy with its main disadvantage: stricture recurrence.^14^

Several methods have been proposed for the prevention of recurrence, such as: long term indwelling Foley catheter up to 6 weeks, intraurethral stents and laser. But there have been no suggestions regarding the use of chemical agents in this area.^15,16,17^ Of course, injection of the stricture site with steroids was tried in the 1960s and 1970s, mainly using Triamcinolone, and little success was reported.^3^ Although Mazdak and his colleagues in 2010 used an intraurethral sub mucosal injection of Triamcinolone in 23 patients after internal urethrotomy and compared them with 22 patients without injection. In their patients urethral stricture recurred in five patients (21.7%) in the triamcinolone group and in 11 patients (50%) in the control group (P = 0.04). They concluded that injection of triamcinolone significantly reduced stricture recurrence after internal urethrotomy.^4^

The antifibroblastic and anticollagenic characteristics of MMC were reported for the first time in the ophthalmology and ENT fields. In one experiment, during trabeculectomy, MMC 0.2mg/cc was applied under the scleral flap for 3 minutes, with a success rate of 83% during first year and 60% during second year. (Success=intraocular pressure<15mmhg).^6,11,18,19^ In another study, MMC was used for post-traumatic subglottic scar successfully without causing any trouble for epithelial regeneration.^20^ Also it has been successfully utilized in myringotomy for otitis media.^7,8,9,10^

To our knowledge, before our study, two other studies have used MMC for the prevention of anterior urethral stricture recurrence. The first study concluded that low dose MMC may be useful for internal urethrotomy in rat model induced fibrosis^21^ in 2nd study, they randomized forty male patients with bulbar urethral strictures to undergo internal urethrotomy with or without urethral sub mucosal Mitomycin C injection. They injected 0.1 mg Mitomycin C submucosally at the planned urethrotomy site using a 22-gauge cystoscopic needle in 20 patients before incising the mucosa; the other 20 patients did not undergo sub mucosal injections. They concluded that sub mucosal injection of Mitomycin C significantly reduced the stricture recurrence rate after internal urethrotomy.^12^

Owing to possible complications from extensive extravasations of MMC through the spongiosum and into the venous drainage and a relatively difficult procedure,^3^ we were encouraged to look for a novel way to apply MMC locally with the best efficacy and as few complications as possible.

MMC (Mitomycin C) is an antibiotic derived from Streptomyces Caespitosus. It contains Quinone, Carbamate and Aziridineeach which probably play a role in its activity. The chemical characteristics of MMC indicate that producing a "ready to use" aqueous formulation of MMC is impossible, even in a refrigerator. But a stable solution of MMC for injection or dilution with water has been produced. It was made by mixing MMC in 40-100% propylene glycol and 0-60% water. The solubility of MMC in water at 25°C is 10 mg/cc but its solubility in a 60-90% mixture of propylene glycol-water is 13-16 mg/cc. Therefore, by appropriate setting of water content in the mixture of MMC/propylene glycol 5-12 mg/cc and water 0-50%, this solution can be kept at 4°C for about 2 years.

Research, especially in the field of biodegradable materials has been performed to promote the control of drug release and elimination of the demand of withdrawal of the non-absorbable drug remnants.^13,22^ Many biodegradable polymers have been tested as matrixes for drugs, such as: polyesters, polycarbonates, synthetic and natural polyamides, polyphosphate esters, polyphosphosines and poly-anhydrides. They have been used in different models: Biodegradable polymer shape-memory stents, "Smart" hydrogel-based systems, nanoparticle-aptamer conjugates and miniaturized drug delivery devices. Many specific smart gels are used, in the urology field, one of which is thermo-reversible Pluronic F-127 Gels.^13,22^

PF-127 is a non-ionic polymer containing polyethylene and polypropylene oxide units. It has a unique specificity for making thermal sensitive gels, solubility of various drugs, slowing the release of the drug and prolonging its efficacy, and its minimal toxicity. Aqueous solutions of this polymer in appropriate densities manifest reverse gelatinization, at 4 to 5°C, they are liquid and with an increase in temperature they transform to gel. To produce this delivery system, "cold method" is used: polymer is added to an aqueous system slowly and at a low temperature, then a drug with a certain dose is added to the system. Then the formulation is injected in a cold environment (4 to 5°C). At body temperature it would create a semi-transparent and semi-solid matrix, which serves as a reservoir for constant slow drug release. This polymer is biodegradable and decomposes to nontoxic elements; therefore there is no need for any extra surgery to remove the remnants.^13,23,24,25^

Furthermore, as there is a high prevalence of urethral stricture and its recurrence and this is a burden on male hygiene, finding newer, more minimally aggressive interventional therapeutic methods should be treated as a priority; thus we were obliged to substitute intraurethral injection by cystoscopic needle and following the policy of reduction of the complications ^26^ and providing appropriate background for the best confrontation of the drug to the targeted tissue, we tried hydrogel-based MMC.

Analysis of our results proved that the drug has reduced stricture recurrence satisfactorily. Although, we used MMC concentration in our medication, eight times more (0.8 vs. 0.1mg) than the concentration which Mazdak and colleagues,^12^ injected in the fibrosis site, we did not see any of side effects related to Mitomycin C. The reason for choosing a higher concentration was to improve the efficacy of hydrogel. Suffice it to say, this method is safe, inexpensive, available, easy to perform, accessible, has few side effects and is non-aggressive. This is probably the first study to introduce hydrogel-based MMC in the prevention of urethral stricture recurrence. However, we have some limitations and disadvantages with this method. First of all, injection must be performed fast (less than 2 minutes), otherwise hydrogel will transform to gel, making its passage through the feeding tube impossible. In addition, it cannot be exactly shown whether the applied jelly is in the stricture area nor how much its effective material is absorbed and how much its absorbed level would be. To better understand these disadvantages, further studies are needed with a longer follow up time and using a contrast agent in solution preparation, with radiologic follow up during postoperative period.

As we mentioned, although we couldn’t prove how much of our medication was absorbed in the stricture area, our result was good, so this study can open the door to using this method for future researchers.

## Conclusion

Based on our results, MMC Hydrogel may have antifibrotic action-preventing anterior urethral stricture recurrence with no side effects on pre-urethral tissue. Due to our study limitations, our follow up time and an insufficient number of patients, further studies are needed with a longer follow up time.

## Acknowledgments

We were not funded by any private institution. The study has been totally conducted by the Kermanshah University of Medical Sciences (KUMS). 
